# Integration of SNP and mRNA Arrays with MicroRNA Profiling Reveals That MiR-370 Is Upregulated and Targets NF1 in Acute Myeloid Leukemia

**DOI:** 10.1371/journal.pone.0047717

**Published:** 2012-10-15

**Authors:** Laura García-Ortí, Ion Cristóbal, Cristina Cirauqui, Elisabet Guruceaga, Nerea Marcotegui, María J. Calasanz, Remedios Castello-Cros, María D. Odero

**Affiliations:** 1 Division of Oncology, Center for Applied Medical Research (CIMA), University of Navarra, Pamplona, Spain; 2 Department of Genetics, University of Navarra, Pamplona, Spain; 3 Unit of Proteomics, Genomics and Bioinformatics, Center for Applied Medical Research (CIMA), University of Navarra, Pamplona, Spain; Emory University, United States of America

## Abstract

**Background:**

Deregulated miRNA expression plays a crucial role in carcinogenesis. Recent studies show different mechanisms leading to miRNA deregulation in cancer; however, alterations affecting miRNAs by DNA copy number variations (CNV) remain poorly studied.

**Results:**

Our integrative analysis including data from high resolution SNPs arrays, mRNA expression arrays, and miRNAs expression profiles in 16 myeloid cell lines highlights that CNV are alternative mechanisms to deregulate the expression of miRNAs in acute myeloid leukemia (AML), and represent a novel approach to identify novel candidate genes involved in AML. We found association between the expression levels of 19 miRNAs and CNVs affecting their loci. Functional analysis showed that NF1 is a direct target of miR-370, and that overexpression of miR-370 has similar effects that NF1 inactivation, increasing proliferation and colony formation in AML cells. Moreover, real time RT-PCR showed that *NF1* downregulation is a recurrent event in AML (30.8%), and western blot analysis confirmed this result. MiR-370 overexpression and deletions affecting the *NF1* locus were identified as alternative mechanisms to downregulate *NF1*.

**Conclusions:**

*NF1* downregulation is a common event in AML, and both deletions in the *NF1* locus and overexpression of miR-370 are alternative mechanisms to downregulate *NF1* in this disease. Our results suggest a leukemogenic role of miR-370 through *NF1* downregulation in AML cells. Since NF1 deficiency leads to RAS activation, patients with AML and overexpression of miR-370 may potentially benefit from additional treatment with either RAS or mTOR inhibitors.

## Introduction

Acute myeloid leukemia (AML) is a heterogeneous clonal disease characterized by enhanced proliferation and impaired differentiation of early progenitors. Its heterogeneity is caused by a variety of genetic and epigenetic aberrations that, acting in combination, contribute to the initiation and progression of this disease. In addition, it has recently been reported the implication of specific microRNAs (miRNAs) in the pathogenesis of AML [1]. MiRNAs are small, non-coding RNAs that bind to the 3′-untranslated region of target genes, negatively regulating their expression levels by translation repression or mRNA degradation. MiRNAs are essential in key biological functions, such as cellular differentiation, development, stress response, apoptosis and cell growth [2]. In addition, miRNAs play important roles in normal hematopoiesis regulating hematopoietic differentiation, and their aberrant expression has been associated with hematological malignancies [1], [3]. Several mechanisms are reported to lead to miRNA deregulation: mutations, chromosomal translocations, epigenetic alterations, or a defective miRNA biogenesis; however, little is known about the mechanisms of miRNA deregulation in AML [2].

MiRNA microarrays in large series of AML cases have identified miRNA signatures associated with several cytogenetic and molecular groups [1]. Furthermore, functional effects of some miRNA alterations have also been reported. For example, miR-155, which shows leukemogenic properties, has been found up-regulated in AML patients with *FLT3*-ITD [4]. In the same way, upregulation of miR-181a and miR-335 has been associated with *CEBPA* mutations and consequently, implicated in the regulation of several genes involved in erythroid differentiation in cytogenetically normal AML (CN-AML) [1]. Interestingly, higher miR-181a expression has been significantly associated with better outcome in CN-AML patients [5].

Analysis of human and mouse genomes reveals that miRNAs are frequently located at fragile sites and regions affected by copy number variations (CNVs) associated with cancer, suggesting that genomic instability could be an important mechanism of miRNA deregulation in cancer [6]. Recently, Starczynowsky et al. identified 18 miRNAs implicated in cellular processes relevant to AML, which map to common leukemia-associated genomic alterations in AML [7]. Here, we analyzed 16 myeloid cell lines using SNP and mRNA arrays, and quantified the expression of 250 mature miRNAs by real-time PCR (QRT-PCR). We identified 19 miRNAs with a significant association between their expression and the CNV of the corresponding genomic region in which the miRNAs were located. This integrative approach, together with bioinformatics and functional studies, allowed us to find that miR-370, located in a recurrent amplified region, was upregulated and that its target gene was the tumor suppressor *NF1*. Interestingly, functional analysis showed that overexpression of miR-370 has similar effects that NF1 inactivation, increasing proliferation and colony formation in AML cells. Finally, overexpression of miR-370 and deletions affecting the *NF1* locus were identified as contributing mechanisms to *NF1* downregulation in AML.

## Results

### Deregulation of miRNAs by gene copy number alterations in AML cell lines

To identify miRNAs deregulated by gene copy number alterations in AML cells, we first performed a SNP array analysis of 16 myeloid cell lines (Table S1 and Table S2). We next analyzed by QRT-PCR the expression profile of 250 miRNAs in these cell lines, evaluating whether the miRNAs located within the amplified or deleted regions identified by the genome-wide analysis were up- or downregulated. Of the 250 miRNAs, 19 showed a significant association between their expression and the CNV of the genomic region in which they were located, and were validated (*P*<0.05). Sixteen miRNAs were upregulated and located in two genomic regions of amplification (11q24.1 and 14q32.31), and 3 miRNAs were downregulated and located in regions with genomic deletions (9p21.32) (Figure S1 and Table 1). Altogether, these results indicate that CNVs are mechanisms that could lead to miRNA deregulation in AML cells.

**Table 1:** MicroRNA deregulated by copy number variations in 16 myeloid cell lines.

MicroRNA	Cytoband	Start	End	Copy number value (mean)	MicroRNA expression (QRT-PCR)	Myeloid cell lines
**miR-7**	9q21.32	85774483	85774592	0.9596	low	EOL-1, NOMO1, F36P, K562, KYO
**miR-15a**	13q14.2	50623255	50623337	1.071	low	F36P, K562
**miR-16-1**	13q14.2	50623109	50623197	1.071	low	F36P, K562
**miR-192**	11q13.1	64415185	64415294	3.365	high	KAS-1, OCI-AML2, F36P, HEL, K562, KU812, KYO, MEG01
**miR-194**	11q13.1	64415403	64415487	3.365	high	KAS-1, F36P, HEL, KG-1, K562, KU812, MEG01
**miR-100**	11q24.1	121528147	121528226	3.099	high	HEL, TF-1, KU812, KYO
**miR-125b**	11q24.1	121475675	121475762	3.099	high	HEL, TF-1, KU812, KYO, MEG01
**miR-370**	14q32.31	121528127	121528246	3.671	high	TF-1, KU812, K562, MEG01
**miR-127**	14q32.31	100419069	100419165	3.671	high	TF-1, KU812, K562, MEG01
**miR-134**	14q32.31	100590777	100590849	3.671	high	TF-1, KU812, MEG01
**miR-154**	14q32.31	100595845	100595928	3.671	high	TF-1, KU812, K562, MEG01
**miR-376**	14q32.31	100576872	100576939	3.671	high	KU812, MEG01
**miR-379**	14q32.31	100558156	100558222	3.671	high	KU812, MEG01
**miR-382**	14q32.31	100590396	100590471	3.671	high	TF-1, KU812, MEG01
**miR-409-5p**	14q32.31	100601390	100601468	3.671	high	TF-1, KU812, MEG01
**miR-432**	14q32.31	100420573	100590471	3.671	high	TF-1, KU812, K562, MEG01
**miR-433**	14q32.31	100417976	100418068	3.671	high	TF-1, KU812, K562, MEG01
**miR-485-5p**	14q32.31	100558156	100558222	3.671	high	TF-1, KU812, MEG01
**miR-494**	14q32.31	100565724	100565804	3.671	high	TF-1, KU812, MEG01

Amp: amplification; Del: deletion.

### Identification of the tumor suppressor NF1 as a target of miR-370

**Figure 1:**
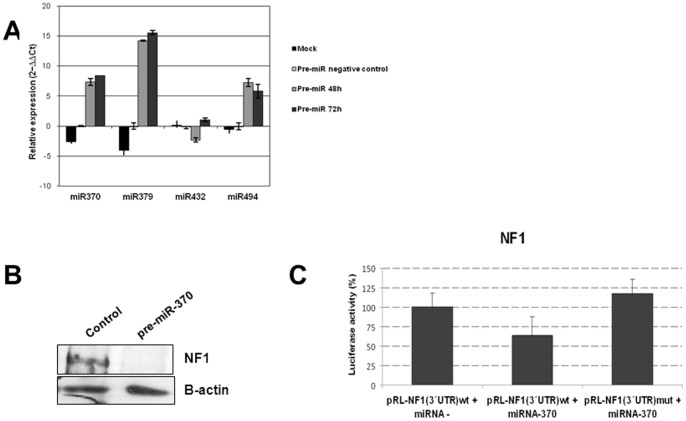
Functional analysis showing that miR-370 regulates *NF1*. (A) miRNAs expression analysis by real-time PCR after transfection with pre-miRs-370, −379, −432 and −494. (B) Western blot showing NF1 after transfection with pre-miR-370. (C) Luciferase assay showing changes in luciferase activity after transfection with pre-miR– (negative control) or pre-miR-370 in cells expressing the 3′UTR region of *NF1* that includes the miR-370 seed region [pRL-NF1(3′UTR)wt]. Transfection with the 3′UTR region of *NF1* including a mutated seed region for miR-370 was used as control.

**Figure 2:**
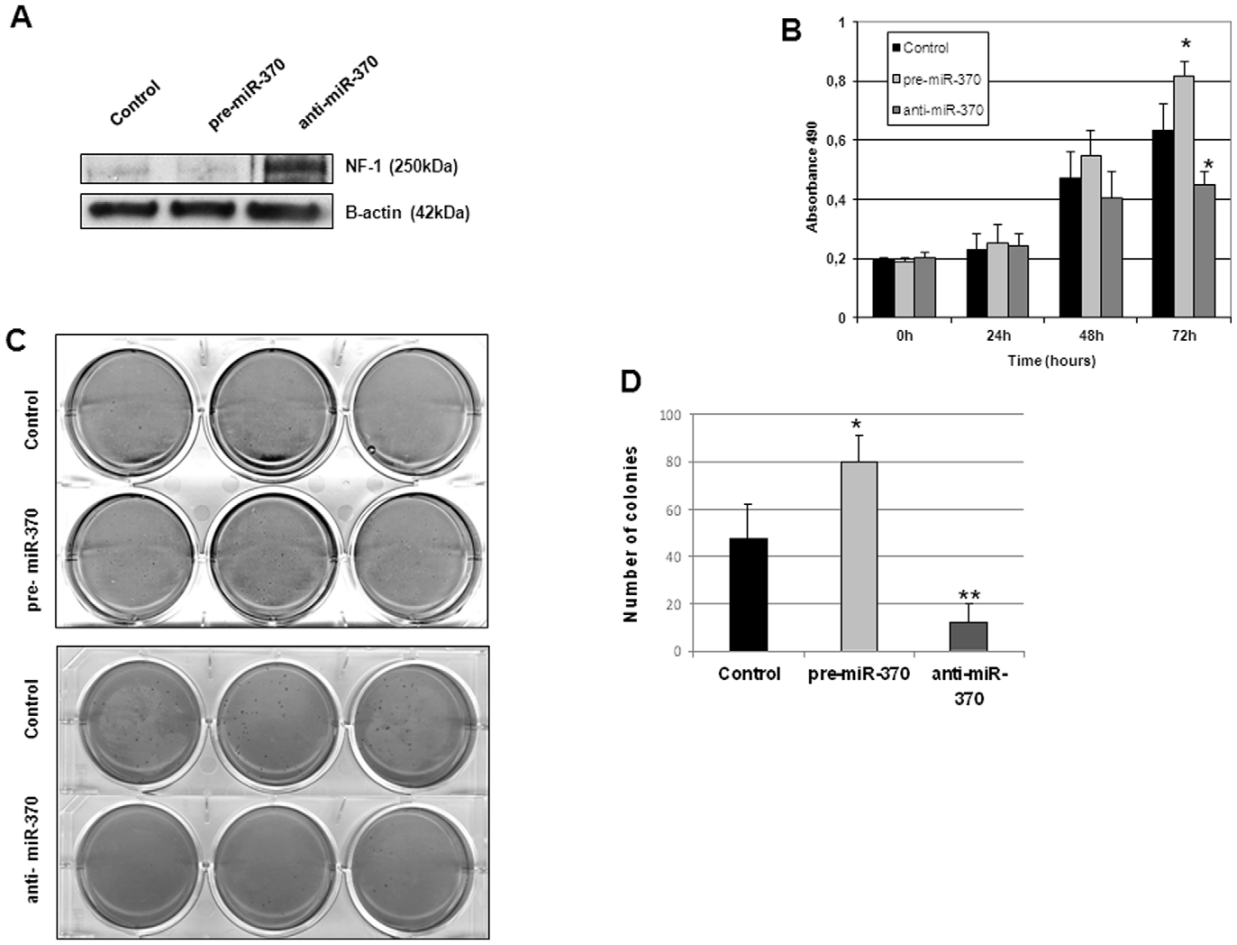
Effects of miR-370 on AML cell proliferation. (A) *NF1* expression after transfection with pre-miR-370 and anti-miR-370 in TF-1 cells. (B) Cell growth of TF1 cells after transfection with pre-miR-370, anti-miR-370 or miR-Control. Bars represent the mean ± SD of three independent experiments. *P<0.05 determined using t-student test. (C) Colony forming ability of TF-1 cells transfected with miR-Control, pre-miR-370 or anti-miR-370. Bars represent the mean ± SD, experiments were done in triplicate. *P<0.05 determined using t-student test.

These results prompted us to determine the potential target genes for those 19 miRNAs whose expression were associated with CN alterations. For this purpose, we performed a whole genome expression analysis in the myeloid cell lines (Affymetrix Human Genome-U133 Plus-2.0). We observed that 4 out of these 19 miRNAs (miR-370, miR-379, miR-432 and miR-494) had *NF1* as a potential target gene (Table S3). Results were validated by QRT-PCR. Therefore, we decided to analyze whether these four miRNAs, all located on 14q32.31, could regulate *NF1*. The AML cell line HL-60, with low expression of the miRNAs and expression of *NF1*, was chosen as a cellular model for miRNA overexpression experiments. QRT-PCR confirmed overexpression of miR-370, miR-379, and miR-494 after transfection with the corresponding pre-miRNAs (Figure 1A). Transfection of premiR-432 could not be optimized. Western blot analysis showed that NF1 levels decreased after miR-370 overexpression (Figure 1B and Figure 2A). No changes in NF1 levels were observed after ectopic expression of miR-379 and miR-494 (data not shown). Transfection of pRL-NF1(3′UTR) in cells ectopically expressing miR-370 showed decreased luciferase reporter activity, indicating that miR-370 binds to the 3′UTR of *NF1*, negatively regulating its expression. Analysis using the same construct with the seed region of miR-370 mutated showed no changes in luciferase activity, confirming that miR-370 directly binds to *NF1* (Figure 1C).

### MiR-370 enhances oncogenic potential of AML cells

It has been reported that *NF1* directly influences AML blast proliferation/growth [14]. Therefore, we first evaluated the functional effects of the transient downregulation of NF1. As expected, we found that NF1 knockdown by siRNA increased cell growth and number of colonies of TF-1 cells as compared to controls (Figure S2). To determine the effects of miR-370 in AML cells, we performed cell growth and colony formation analysis in TF-1 cells transfected with pre- and anti-miR-370. Downregulation of NF1 by miR-370 and upregulation by anti-miR-370 in these cells were confirmed by western blot (Figure 2A). We first analyzed the effects on cell growth of miR-370 and anti-miR-370 using a MTS assay. Consistent with the tumor suppressor activity of *NF1*, there was increased proliferation in TF-1 cells transfected with pre-miR-370 in comparison with control cells (Figure 2B). Overexpression of miR-370 was confirmed by QRT-PCR. Conversely, ectopic expression of anti-miR-370 significantly reduced cell growth of these cells (Figure 2B). To further confirm the importance of miR-370 as regulator of AML cell proliferation, we determined the effects on colony-forming ability of miR-370 in AML cells. Pre-miR-370-transfected TF-1 cells formed significantly higher number of colonies than controls, while anti-miR-370 reduced the number of colonies (Figure 2C). It was not possible to confirm these results in HL-60 because this cell line has an activating mutation in NRAS. Altogether, these results would suggest a leukemogenic role of miR-370 through *NF1* downregulation in AML cells.

### NF1 downregulation is a recurrent event in AML

To further evaluate the clinical relevance of *NF1* in AML, we quantified *NF1* expression by QRT-PCR in a series of 68 patients with AML at diagnosis. Patient characteristics are shown in Table 2. *NF1* was downregulated in 30.8% cases (21/68). Levels of miR-370 could be determined in 50 out of these 68 patients, and we observed that miR-370 was overexpressed in 12% cases (6/50). *NF1* was downregulated in all the 6 cases with miR-370 overexpression, indicating a good association between both aberrations (Figure S3 and Table S4). To investigate other mechanisms involved in *NF1* downregulation, we analyzed the copy number at the *NF1* locus by Q-PCR in 55 out of the 68 cases. We found that 13 patient samples had deletions affecting *NF1* (13/55, 23.6%) (Table 2 and Table S4). These findings indicate that copy number changes involving the *NF1* locus represent a mechanism contributing to *NF1* deregulation in AML cells. Interestingly, 5 out of the 6 cases with overexpression of miR-370 had normal number of copies of *NF1* (Table S4), indicating that they are alternative mechanisms of *NF1* downregulation in AML. Altogether, these results suggest that *NF1* downregulation is a recurrent event in AML, and that both miR-370 overexpression and submicroscopic deletions of the *NF1* locus may represent important mechanisms to downregulate *NF1* expression in AML patients.

**Table 2:** Clinical and molecular characteristics of the 70 patients with AML at diagnosis included in the study.

Sex		No.	(%)
	Male	49	(70)
	Female	21	(30)
Age			
	<60 years	24	(34.8)
	≥60 years	45	(65.2)
	No data	1	
Diagnosis			
	AML-M0	6	(8.6)
	AML-M1	19	(27.2)
	AML-M2	16	(22.8)
	AML-M4	6	(8.6)
	AML-M5	15	(21.4)
	AML-M6	2	(2.8)
	AML-NOS	6	(8.6)
Secondary AML			
	No	60	(87)
	Yes	9	(13)
	No data	1	
Cytogenetic group			
	good	4	(5.8)
	intermediate	59	(85.5)
	poor	6	(8.7)
	No data	1	
*NF1* downregulation			
	No	47	(69.2)
	Yes	21	(30.8)
	No data	2	
Deletions affecting *NF1* locus			
	No	32	(76.4)
	Yes	13	(23.6)
	No data	15	
miR-370 overexpression			
	No	40	(87)
	Yes	6	(13)
	No data	24	
*FLT3*-ITD			
	No	40	(78.4)
	Yes	11	(21.6)
	No data	19	
NPM1 mutated			
	No	23	(54.8)
	Yes	19	(45.2)
	No data	28	

**Figure 3:**
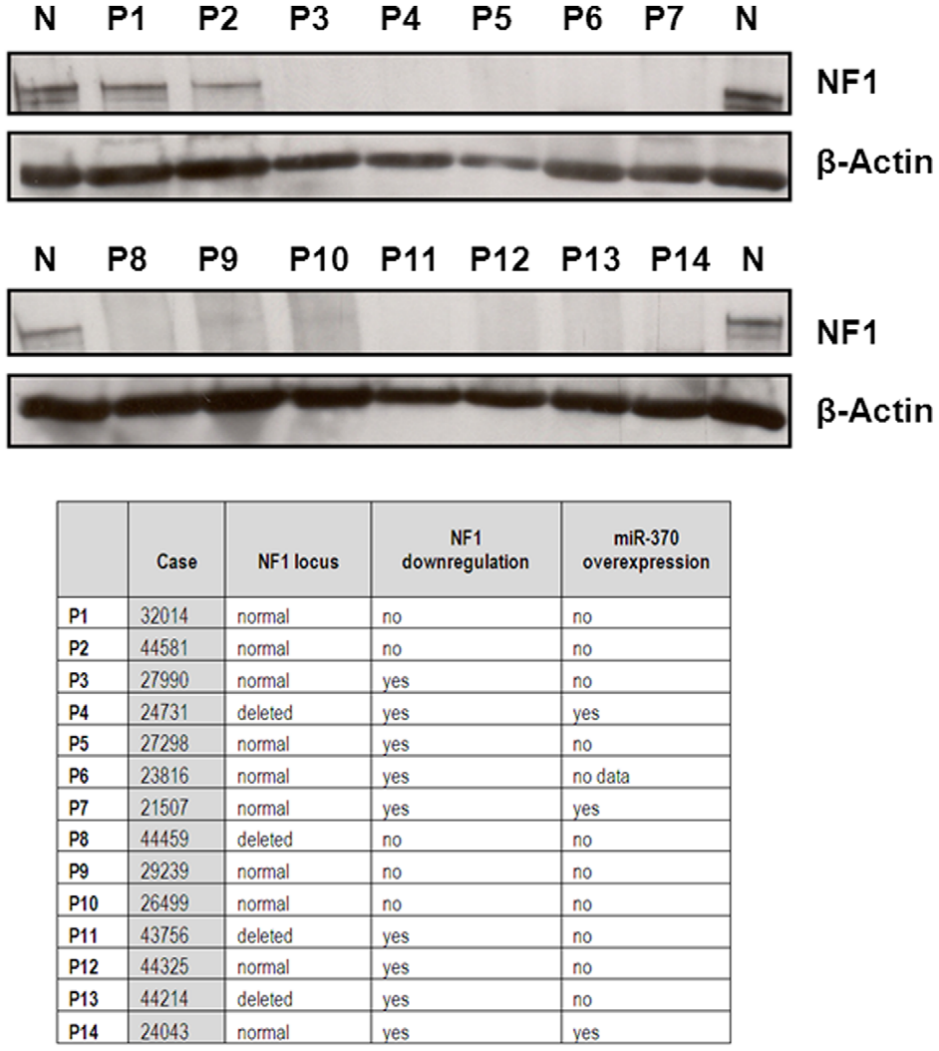
Western blot analysis of NF1 in 14 samples of patients with AML at diagnosis. Data about NF1 status and expression of miR-370 are shown. N: normal control; P: patient sample.

To confirm these results at protein level, we analyzed NF1 by western blot in 14 cases with AML at diagnosis: 9 had *NF1* downregulation and 5 normal mRNA levels of *NF1* (Figure 3). Patient characteristics are included in Table S5. NF1 protein was not detected in any of the 9 cases with *NF1* downregulation, including the 3 cases with miR-370 overexpression (P4, P7, and P14). Moreover, one of the five patients with normal mRNA levels of *NF1* had no protein (case P8), and other two cases had reduced expression of the NF1 protein (cases P9 and P10).

## Discussion

Although the association of miRNA expression profiles with cytogenetic or molecular aberrations has been widely investigated in AML [1], [20], the relevance of copy number changes affecting genomic regions that include miRNAs remains poorly explored. In this study, we show that CNVs are alternative mechanisms that regulate the levels of some miRNAs, which, in turn, modulate the expression of target genes with importance in AML development. Using an integrative analysis based in SNP, mRNA arrays and expression profile of 250 mature miRNAs, together with bioinformatics and functional studies, we found that miR-370, located in a recurrent amplified region, was upregulated and that its target gene was the tumor suppressor *NF1*. Interestingly, functional analysis showed that overexpression of miR-370 has similar effects that *NF1* inactivation, suggesting a leukemogenic role of miR-370 through *NF1* downregulation in AML cells. Besides, our results show that *NF1* downregulation is a common event in AML, and that both deletions in the *NF1* locus and overexpression of miR-370 are mechanisms involved in this downregulation.

Two recent studies in series of AML patients showed that microdeletions of *NF1* are common events in this disease, leading to reduction of *NF1* expression [14], [21]. Importantly, we describe here a novel regulatory mechanism by which miR-370 modulates *NF1* expression by directly binding to its 3′-UTR. Germline loss-of-function mutations of *NF1* lead to neurofibromatosis type 1, a dominant autosomal genetic disorder clinically characterized by neurofibromas, cafe-au-lait spots, and a high risk to develop juvenile myelomonocytic leukemia (JMML) [22]. The *NF1* gene protein product, neurofibromin, is a GTPase-activating protein (GAP) that inhibits RAS signaling by hydrolysis of active RAS-GTP into inactive RAS-GDP; therefore, *NF1* deficiencies act as functional equivalents of activating mutations in *RAS*. The finding that the wild-type allele was lost in the bone marrow of children with JMML affected by neurofibromatosis type 1 established *NF1* as a tumor suppressor gene [23]. In fact, somatic inactivation of *NF1* in hematopoietic cells results in a progressive myeloproliferative disorder in mice, with elevated levels of RAS-GTP [24], although secondary genetic events are required to the development of AML. Recently it was shown that *NF1* downregulation in AML blasts caused a substantial and significant increase in AML blast colony formation in methylcellulose, confirming that *NF1* directly influences AML blast proliferation/growth [14]. Our functional studies confirmed these results and showed that overexpression of miR-370 promoted cell growth and colony forming potential of AML cells, the same effects that *NF1* downregulation. Altogether, these data strongly suggest that overexpression of miR-370 represents a novel mechanism of *NF1* inactivation in AML, and that this microRNA plays a relevant role in AML proliferation by downregulating *NF1* expression. It has been reported that miR-370 is overexpressed in acute promyelocitic leukemia [25] and gastric carcinomas [26]. However, contradictory results have been described about the functional role of this microRNA. Meng et al. found that miR-370 is a methylation-dependent miRNA regulated by interleukin-6 (IL-6), and that overexpression of miR-370 reduced the growth of cholagiocarcinoma cells by targeting the oncogene mitogen-activated protein kinase 8 (MAP3K8) [27]. In contrast, Lo et al. described that miR-370 enhanced the oncogenic potential of gastric carcinoma (GC) [26]. However, although they found that the increased in mobility induced by miR-370 in GC cells was via targeting transforming growth factor-β receptor II (TGFβ-RII), they did not observe any influence of the TGFβ pathway in the enhanced tumor cell growth elicited by miR-370 in GC cells. According to our results, it is possible that the enhanced growth after overexpressing miR-370 in GC cells observed by these authors was mediated by downregulation of *NF1*. The opposite roles of miRNA-370 in different malignancies could be explained considering that microRNAs are able to regulate the expression of multiple genes. Therefore, it is possible that miR-370 modulates the expression of all these targets; however, the dominant event may depend on cell type or cellular contexts [28]. Although miR-370 is likely to target other genes in addition to *NF1*, downregulation of *NF1* appears to play a critical role in enhanced tumorigenicity associated with overexpression of miR-370 in AML.

Quantification of the expression of *NF1* in our collection of 68 samples of patients with AML at diagnosis showed that *NF1* downregulation is a recurrent event in AML, accounting for 30.8% cases. Parkin et al. have also analyzed *NF1* expression by QRT-PCR in AML cases, and showed in their Figure 2 the high frequency of *NF1* downregulation in patients with one as well as with two copies of *NF1*; however, they did not indicate the number of cases with this downregulation [14]. Therefore, our results showed for the first time the prevalence of *NF1* downregulation in AML by QRT-PCR. Further studies in larger series of cases are needed to establish the prevalence of NF1 downregulation in AML. Deletions or/and mutations affecting *NF1* have been described as genetic aberrations responsible for *NF1* inactivation in adult AML, although inactivation mutations of *NF1* are rare events in de novo AML. Parkin et al. reported a detailed investigation in 95 AML patients and showed that 10 out of 95 (10.5%) had heterozygous deletions of the *NF1* locus, a lower percentage than our study (13/55, 26.5%)[14]. Since we used the same technique reported by this group, these results would indicate that additional studies are required to determine the real prevalence of *NF1* submicroscopic deletions in AML. In addition, in a recent study, Haferlach et al. showed that the prevalence of NF1 mutations in AML is 2% (19/889) [21]. In our study, we found 21 cases with reduced *NF1* at mRNA level: 5 had *NF1* deletions, 6 miR-370 overexpression, and 11 none of these aberrations (one case had both *NF1* deletion and overexpression of miR-370). Therefore, our results confirm the studies that showed that CNV could be one of the causes of *NF1* downregulation in AML [14], [21], identify overexpression of miR-370 as a new mechanism of *NF1* downregulation in AML, and suggest that there are other unknown mechanisms involved in *NF1* downregulation. Therefore, it is possible that the altered expression of miR-370 could explain the 3 cases identified by Parkin et al. with normal copy number and null *NF1* expression [14].

Furthermore, in this study we have analyzed for the first time the status of NF1 protein in 14 AML patient samples, showing that the finding of null *NF1* is frequent in AML. Lu et al. (2003) had previously reported decreased bone marrow levels of the NF1 protein in 30% cases with AML (20/66) (24), a prevalence similar to that observed in this study at mRNA level (30.8%) (Table 2). The authors used solid-phase RIA to measure the NF1 protein because they were not able to detect NF1 by western blot in bone marrow samples from normal individuals. We could detect the NF1 protein in normal controls, suggesting that our results in AML cases with downregulation of Nf1 at protein level are reliable. Importantly, our results show that all patients with *NF1* downregulation, including 3 cases with overexpression of miR-370, have no protein at all. Moreover, we identified a case with normal levels of both *NF1* mRNA and miR-370 and no protein, confirming the complex regulation of *NF1*, and showing that there are other mechanisms of *NF1* regulation at post-transcriptional level. Parkin at al. have shown that complete *NF1* loss is required to Ras activation, and that heterozygous *NF1* states that preserve some *NF1* expression are not sufficient for robust Ras activation [14]. Of note, AML blasts without functional *NF1* were substantially and significantly more sensitive to mTOR inhibition than *NF1* wild-type blasts or blasts with one preserved *NF1* copy and retained *NF1* expression [14]. Therefore, our results would point out to a new group of patients susceptible of receiving these therapies.

In summary, our integrative analysis including data from high resolution SNPs arrays, mRNA expression arrays, and miRNAs expression profiles in 16 myeloid cell lines highlights that copy number alterations are alternative mechanisms to deregulate the expression of miRNAs in AML, and represent a novel approach to identify novel candidate genes involved in AML. Of note, functional studies identified *NF1* as a target of the miR-370 and, in agreement with the tumor suppressor activity of *NF1*, we found that overexpression of miR-370 enhanced the tumorigenic potential of AML cells. Finally, QRT-PCR and western blot analyses showed that *NF1* downregulation is a common event in AML, and that both deletions in the *NF1* locus and overexpression of miR-370 represent two alternative mechanisms to downregulate *NF1* in this disease. Since *NF1* deficiency leads to the activation of the RAS signaling pathway, patients with AML and overexpression of miR-370 may potentially benefit from additional treatment with either RAS or mTOR inhibitors. Further studies are required to identify other mechanisms leading to *NF1* deficiency and to decipher the prognostic impact of *NF1* inactivation in AML.

## Materials and Methods

### Cell culture and transfection

EOL-1, HL-60, Kasumi-1, OCI-AML2, MOLM13, MV4-11, HEL, KG-1, KYO-1, K562 and MEG-01 cells were maintained in RPMI-1640 (Invitrogen) with 10% fetal bovine serum (FBS); NOMO-1 and KU-812 in RPMI-1640 with 20% FBS; F-36P in RPMI-1640 with 20% FBS, and 10 ng/ml GM-CSF; MUTZ-3 in 80% alpha-MEM with 20% FBS and 10 ng/ml GM-CSF; and TF-1 in RPMI-1640 with 20% FBS and 5 ng/ml GM-CSF. Cell lines were grown at 37°C in a 5% CO2 atmosphere. Media were supplemented with penicillin G (100 U/ml), and streptomycin (0.1 mg/ml). Cell lines were obtained from the DSM Cell Culture Bank (Braunschweig, Germany). Characteristics of the 16 myeloid cell lines used in this study are summarized in Table S6. For transfection experiments HL-60 and TF-1 cells were seeded in culture flasks and transfected using the Nucleofector System (solution V and protocol T-019 for HL-60 cells; solution T and protocol T-001 for TF-1 cells) (Amaxa) with 5 nM of pre-miRNAs designed and synthesized by Ambion (Applied Biosystems) (hsa-miR-370 ID/PM12868; hsa-miR-432 ID/PM10838; hsa-miR-379 ID/PM10316; hsa-miR-494 ID/PM12409; anti-hsa-miR-370 ID/AM12868, hsa-miR-Control ID/AM17110), or with 100 pmol of NF-1 and scramble siRNA oligonucleotides (NF-1 siRNA: 5′-AAGGUUGCGCAGUUAGCAGUU-3′ and scramble siRNA: 5′-UUCUCCGAACGUGUCACGU-3′) synthesized by MGW Biotech (Ebersberg, Germany).

### Patient samples

The study comprised bone marrow samples of 70 patients with AML at diagnosis. Bone marrow samples of normal healthy donors were used as controls.

### Ethics Statement

The study has been approved by the Comisión de Ética de Investigación de la Facultad de Medicina de la Universidad de Navarra (037/2008). Informed consent for this study is not required because the samples are anonymous (anonymous samples have neither personal data nor individual clinical information that could allow the identity of the donor to be traced).

### Single Nucleotide Polymorphism Array Analysis

Whole genome analyses were performed using the GeneChip Mapping 500 K Array Set (Affymetrix). Genomic DNA samples were isolated using QiAmpDNA MiniKit (Qiagen). Arrays were scanned individually using the GeneChip® Scanner 3000 7G under the GeneChip® CEL files were generated using Affymetrix GeneChip Command Console operating software and Genotyping Console 2.1 according to the manufacturer protocols (Affymetrix). We only analyzed samples which met the quality control (QC) thresholds recommended by Affymetrix in their Genotyping Console v2.1 software. The QC call rate of samples analyzed was at least 96%. Samples not meeting this specification were excluded from further analysis. CEL files were then imported into Partek Genomic Suite and analyzed using the Copy Number Analysis workflow. Regions of CNVs were detected using an unpaired analysis and Genomic segmentation algorithm in the standard Partek. Genomic alterations identified by SNP array (SNPa) were compared with the 500K HapMap Genotype Data Set. We consider as amplification the regions whose copy number was over 3 copies, and as deletion, regions with copy number below 1.5 (homozygous deletion CN<0.5 and hemizygous deletions CN = 0.8–1.5) [8]. We considered the karyotype of each cell line to asses that results obtained in the SNPa analysis are not due to the presence of a previously described cytogenetic aberration.

### Validation of copy number alterations

Genomic regions located within miRNAs genes were amplified with the primers included in Table S7. Glucose-6-Phosphate Dehydrogenase (*G6PDH*), Hydroxymethylbilane Synthase (*HEM3*) and Chloride channel 7 (*CLCN7*) genes were selected as internal controls for varying input DNA amounts as recommended by prior published guidelines [9], [10]. Thus, any difference in the real time PCR obtained for test primers/markers would correspond to differences in the amount of the target sequence primers. SYBR Green I real time PCR assays were carried out in final reaction volumes of 15 µl with 7.5 µl of SYBR Green I Master mix (Applied Biosystems), 1 µM of forward and reverse primers and 10 ng of genomic DNA. Real time PCR reactions were performed using the 7500 Real Time PCR System (Applied Biosystems). The reaction profile was: initial step, 50°C for 2 min, denaturation, 95°C for 10 min, then 40 cycles of denaturing at 95°C for 15 sec, combined annealing and extension at 60°C for 30 sec and 72°C 30 sec, followed by the dissociation stage of 72°C 10 min. Data were analyzed as previously described [10].

### Quantification of miRNA expression levels

Total RNA was isolated using TRIzol Reagent (Invitrogen) according to manufacturer's instructions. For quantification of miRNA expression levels, samples were reverse transcribed using the TaqManHMicroRNA Reverse Transcription Kit (P/N 4366597, Applied Biosystems) and mature miRNAs were quantified by quantitative real-time RT-PCR (QRT-PCR) using the TaqManH MicroRNA Assays - Human Panel Early Access Kit (P/N 4365409, Applied Biosystems). The kit contained assays for 250 miRNAs of the 733 currently listed in the Sanger miRBase database. Expression levels of miR-370, miR-379, miR-494, and miR-432 were confirmed using TaqMan MicroRNA Assays (Applied Biosystems) specific for each miRNA and U6B as internal control. Analysis of relative gene expression data was performed using the 2^−ΔΔ*C*^_T_ method [11]where ΔΔ*C*_T_ =  (*C*_T,Target Gene_ – *C*_T,U6B_)_Cell Line_ – (*C*_T,Target Gene_ – *C*_T,U6B_)_Normal Control_.

### Gene Expression Profiling

Total RNA was extracted from cell lines using miRNEasy Mini Kit (Qiagen) following manufacturer's protocol. The RNA integrity was assessed using Agilent 2100 Bioanalyzer (Agilent). Whole genome expression analysis was performed in the cell lines using the Affymetrix Human Genome-U133 Plus-2.0, which contains 54,676 probesets (47,000 transcripts). Microarray data analysis consisted in background correction and normalization using RMA algorithm [12] and a filtering process to eliminate low expression probesets. LIMMA (Linear Models for Microarray Data) [13] was used to identify the probesets with significant differential expression. Genes were selected as significant using a *B* statistic cut off (*B*>0) or a less stringent *P*-value cut off (*P*<0.001).

### Quantification of NF1 by real-time RT-PCR and validation of copy number alterations

Total RNA was isolated using the RNeasy minikit (Qiagen). cDNA was synthesized with SuperScriptIII Reverse Transcriptase (Invitrogen). Quantification of the expression of *NF1* was performed by SYBR Green I real time PCR assays (Applied Biosystems), using specific primers for each gene (NF1_Fwd: AAGCCCTCACAACA-ACCAAC; NF1 Rv: GACAATACACAGCATCAATCT; HPRT Fwd: TGACAC-TGGCAAAACAATGCA; HPRT Rv: GGTCCTTTTCACCAGCAAGCT). *HPRT* was used as internal control. Analysis of relative gene expression data was performed using the 2^−ΔΔ*C*^_T_ method [11]where ΔΔ*C*_T_ =  (*C*_T,Target Gene_ – *C*_T,HPRT_)_Cell Line_ – (*C*_T,Target Gene_ – *C*_T,HPRT_)_Normal Control_. A gene was considered deregulated if its expression value was higher or lower than the cut-off value established for each gene (mean+3SD), defined by the analysis of 10 normal BM samples.

Quantification of genomic copy number changes at the *NF1* locus (17q11) was performed using specific TaqMan-based probes for *NF1* (Hs06413068_cn) and *RAG2* (Hs01851142_s1) (Applied Biosystems) as previously described [14]. DNA was extracted using QuiAmp DNA Mini Kit (Qiagen). Analysis of relative gene copy number data for *NF1* was performed using the ΔΔ*C*_T_ method. As indicated by Parkin et al. (2010), the *C*_T_ values for the *RAG2* locus were used as reference [14].

### Integrative Genomic Analyses

Relationship between miRNA expression and the CN of the corresponding miRNA gene locus was measured by a parametric analysis. Data from SNP and miRNA analysis were crossed and analyzed using t-tests. A statistical hypothesis test was performed for each miRNA profile using the results of the CNV analysis (amplification/deletion) as sample labels. Associations were considered statistically significant when P<0.05. The set of differentially expressed genes were further studied using miRNA predicted targets stored in public databases to identify coherent targets [15], [16], [17], [18], [19].

### Western blot analysis

Protein extracts were isolated using TRIzol Reagent (Invitrogen) following manufacturer's indications, clarified (12,000×g, 15 minutes, 4°C), denatured and subjected to sodium dodecyl sulfate-polyacrylamide gel electrophoresis and Western blot. Antibodies used were rabbit polyclonal anti-Nf1 (Santa Cruz) and mouse monoclonal anti-β-actin (Sigma-Aldrich). Proteins were detected with the appropriate secondary antibodies by chemiluminescence (ECL kit, GE Healthcare).

### Luciferase assays

Luciferase assays were done using the Dual Luciferase System (Promega). One hundred nanograms of pRL-NF1(3′UTR) or pRL were transfected in the presence of 5 ng of pre-miR-370 or pre-miR-negative control, and 50 ng of pGL3-Promoter. A pRL-NF1(3′UTR) construct including a mutated miR-370 seed region was used to confirm *NF1* as a direct target of miR-370. Renilla luciferase activities were normalized to firefly luciferase activities.

### Proliferation assays

TF-1 cells transfected with pre-miR-370, anti-miR-370, pre-miR-control, NF-1 siRNA or scramble siRNA, were seeded at 7.5×10^3^ cells/well in 96-well plates. Cell growth was assessed by MTS assay using the CellTiter 96 Aqueous One Solution Cell Proliferation Assay (Promega) and following the manufacturer's indications. Experiments were performed in triplicate and repeated at least 3 times.

### Colony-forming assay

Experiments were performed in 6-well plates were coated with 0.6% soft agarose (Sigma) in medium. 1×10^5^ TF-1 transfected cells were suspended in 0.3% agarose in medium and plated in triplicated over the pre-coated wells. Fresh medium was supplied thrice a week. After 7 days, colonies were stained by adding 500 ul of 5 mg/mL MTT (Methylthiazolyldiphenyl-tetrazolium bromide, M-5655, Sigma) for 4 h at 37°C. Then, colonies were fixed by adding DMSO overnight at 37°C. Colony numbers were determined from triplicates.

## Supporting Information

Figure S1 **Boxplots of miRNAs whose expression significantly correlated with the inferred CN of the corresponding region.** Cell lines are classified as carrying or not **A.** gain/amplification (inferred CN >3) or **B.** loss/deletion (inferred CN <1.5) of each specific miRNA gene.
(TIF)

Figure S2 **Knockdown of NF1 reduces cell growth and colony-forming ability of TF-1 cells.** **A.** Relative NF1 gene expression levels in TF-1 cells 48 h after transfection with scrambled and NF1 siRNA. Bars represent the fold change calculated by the 2−ΔCt method. Expression was normalized to the HPRT1 gene. **B.** Western blot showing NF1 expression levels from TF-1 cells transfected with NF1-targeting or scrambled siRNA, 48 h post-transfection. β-actin was used as a loading control. **C.** Growth curves of scrambled and NF1 siRNA-transfected TF-1 cells. *P<0.05 Student's t test. Data shown are mean ± SD of triplicate cultures and are representative of three independent experiments. **D.** Representative images of colonies formed by scramble and NF1 siRNA-transfected TF-1 cells after two weeks grown in soft agar. **E.** Number of colonies formed in the colony formation assay. **P<0.01, Student's t test. Data represented are mean ± SD.
(TIF)

Figure S3 **Scatterplot showing the significant association between down expression of NF1 and high expression of miR-370.**
(TIF)

Table S1 **Genomic regions with amplifications or deletions found at least in 4 out of the 16 myeloid cell lines analyzed.** Amplification was considered if CN >3, and deletion if CN <1.5.
(DOC)

Table S2 **List of the high amplifications (CN>5) and homozygous deletions (CN<0.5) found in the copy number analysis of 16 myeloid cell lines.**
(DOC)

Table S3 **MicroRNAs located in amplified regions in myeloid cell lines, which were highly expressed, and had** ***NF1*** **as a potential target gene.**
(DOC)

Table S4 **NF1 status and miR-370 expression in the 68 samples of patients with AML at diagnosis included in the study.**
(DOCX)

Table S5 **Clinical and molecular characteristics of 14 patients with AML at diagnosis included in the study of NF1 at protein level.**
(DOC)

Table S6 **Clinical and molecular characteristics of the 16 human myeloid cell lines.**
(DOCX)

Table S7 **Oligonucleotide primers used for real-time Q-PCR.**
(DOC)
