# Supplementary figures and images for: Integration of SNP and mRNA Arrays with MicroRNA Profiling Reveals That MiR-370 Is Upregulated and Targets NF1 in Acute Myeloid Leukemia
Source: PLoS One. 2012 Oct 15;7(10):e47717. doi: 10.1371/journal.pone.0047717 (PMC3471844; doi:10.1371/journal.pone.0047717)

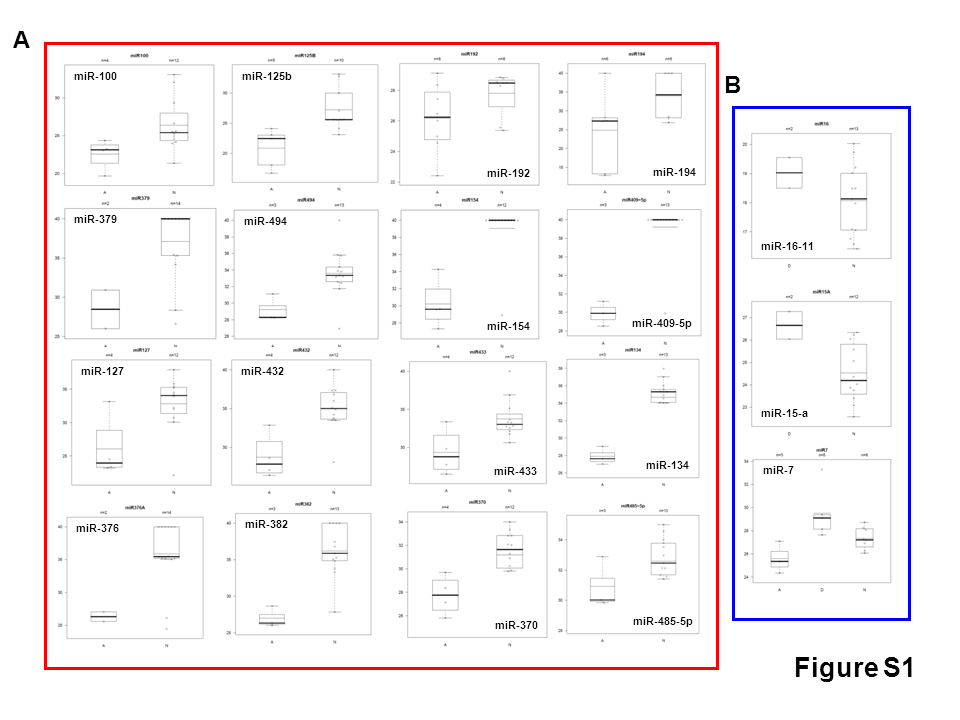

Supplement: Figure S1 — Boxplots of miRNAs whose expression significantly correlated with the inferred CN of the corresponding region. Cell lines are classified as carrying or not A. gain/amplification (inferred CN >3) or B. loss/deletion (inferred CN <1.5) of each specific miRNA gene. (TIF) [file pone.0047717.s001.tif]

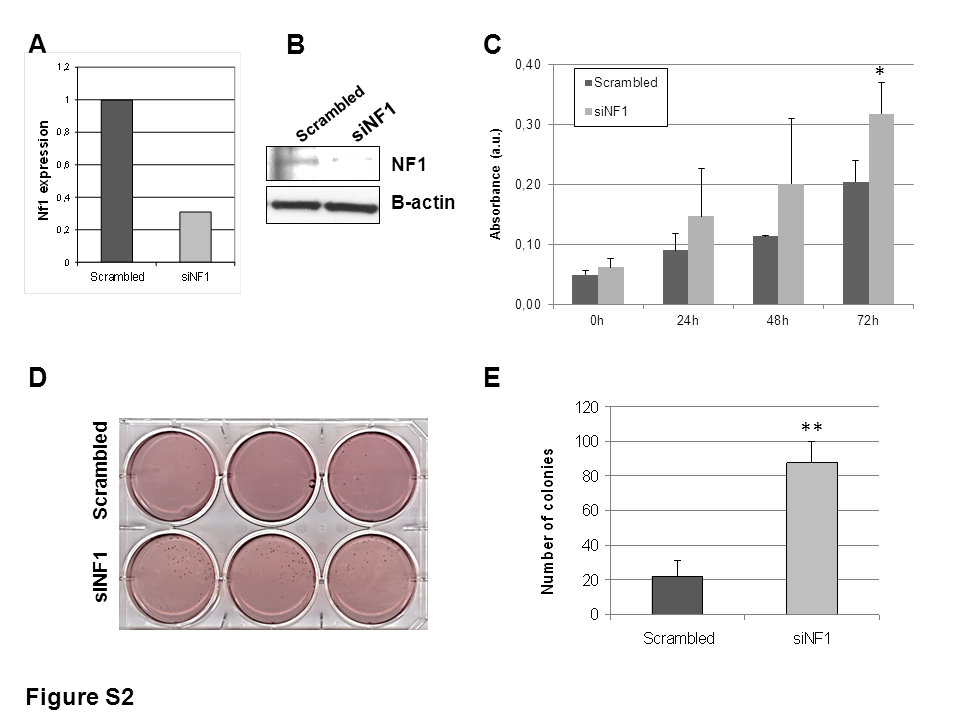

Supplement: Figure S2 — Knockdown of NF1 reduces cell growth and colony-forming ability of TF-1 cells. A. Relative NF1 gene expression levels in TF-1 cells 48 h after transfection with scrambled and NF1 siRNA. Bars represent the fold change calculated by the 2−ΔCt method. Expression was normalized to the HPRT1 gene. B. Western blot showing NF1 expression levels from TF-1 cells transfected with NF1-targeting or scrambled siRNA, 48 h post-transfection. β-actin was used as a loading control. C. Growth curves of scrambled and NF1 siRNA-transfected TF-1 cells. *P<0.05 Student's t test. Data shown are mean ± SD of triplicate cultures and are representative of three independent experiments. D. Representative images of colonies formed by scramble and NF1 siRNA-transfected TF-1 cells after two weeks grown in soft agar. E. Number of colonies formed in the colony formation assay. **P<0.01, Student's t test. Data represented are mean ± SD. (TIF) [file pone.0047717.s002.tif]
