# Supplementary material for: Integration of SNP and mRNA Arrays with MicroRNA Profiling Reveals That MiR-370 Is Upregulated and Targets NF1 in Acute Myeloid Leukemia
Source: PLoS One. 2012 Oct 15;7(10):e47717. doi: 10.1371/journal.pone.0047717 (PMC3471844; doi:10.1371/journal.pone.0047717)
Supplement: Table S1 — Genomic regions with amplifications or deletions found at least in 4 out of the 16 myeloid cell lines analyzed. Amplification was considered if CN >3, and deletion if CN <1.5. (DOC) [file pone.0047717.s004.doc]

**Supplementary Table 1.** Genomic regions with amplifications or deletions found at least in 4 out of the 16 myeloid cell lines analyzed. Amplification was considered if CN > 3, and deletion if CN < 1.5.

|  | **Cell line** | **Cytoband** | **Start** | **End** | **Length (bps)** | **Copy number value** | **Markers** |
| --- | --- | --- | --- | --- | --- | --- | --- |
|  |  |  |  |  |  |  |  |
| A | EOL-1 | 1q32.1 | 201571229 | 201606873 | 35644 | 3,18 | 28 |
|  | Kasumi-1 | 1q32.1 | 203711819 | 203752179 | 40360 | 4,45 | 15 |
|  | KU-812 | 1q32.1 | 199892235 | 199924702 | 32467 | 4,38 | 10 |
|  | MEG-01 | 1q32.1 | 199892235 | 199924702 | 32467 | 3,91 | 10 |
|  | K562 | 1q32.2 - q42.1 | 199100957 | 223701729 | 24600772 | 3,56 | 4837 |
|  | NOMO-1 | 1q32.1 | 203232845 | 203253176 | 20331 | 4,14 | 13 |
|  | MOLM13 | 1q32.1 | 201415031 | 201439334 | 24303 | 3,42 | 10 |
| A | EOL-1 | 3q21.2 | 127326135 | 127350448 | 24313 | 4,86 | 10 |
|  | Kasumi-1 | 3q21.2 - 3q21.3 | 127326135 | 128259831 | 933696 | 6,27 | 20 |
|  | HEL | 3q21.2 - 3q21.3 | 127326135 | 129002970 | 1676835 | 3,60 | 193 |
|  | NOMO-1 | 3q21.2 - 3q21.3 | 127428799 | 165271748 | 37842949 | 3,66 | 5926 |
|  | OCI-AML2 | 3q21.2 - 3q21.3 | 127326135 | 128259831 | 933696 | 4,22 | 20 |
| A | KYO-1 | 11q13.1 | 63637120 | 63855796 | 218676 | 3,39 | 20 |
|  | K562 | 11q12.2 - q13.5 | 60265161 | 75596147 | 15330986 | 3,38 | 1865 |
|  | NOMO-1 | 11q13.1 | 63628813 | 63684339 | 55526 | 4,55 | 10 |
|  | KU-812 | 11q13.1 - q13.5 | 63725602 | 75596147 | 11870545 | 3,82 | 1422 |
|  | F-36P | 11q13.5 | 75544219 | 75596147 | 51928 | 4,17 | 15 |
|  | EOL-1 | 11q13.5 | 75544219 | 75596147 | 51928 | 3,88 | 15 |
|  | Kasumi-1 | 11q13.5 | 75544219 | 75596147 | 51928 | 4,61 | 15 |
|  | KG-1 | 11q13.5 | 75567031 | 75644651 | 77620 | 3,94 | 11 |
|  | MEG-01 | 11q13.5 | 75567031 | 75644651 | 77620 | 5,30 | 11 |
|  | HEL | 11q13.5 | 75544219 | 75584526 | 40307 | 4,05 | 13 |
|  | OCI-AML2 | 11q13.5 | 75544219 | 75584526 | 40307 | 3,68 | 13 |
| A | KG-1 | 16q13 | 55431557 | 55496746 | 65189 | 3,94 | 11 |
|  | KYO-1 | 16q13 | 55431557 | 55496746 | 65189 | 3,72 | 11 |
|  | MEG-01 | 16q13 | 55431557 | 55498995 | 67438 | 3,35 | 13 |
|  | NOMO-1 | 16q13 - q21 | 56561169 | 56714732 | 153563 | 3,62 | 13 |
| A | Kasumi-1 | 20q13.33 | 58939610 | 58965922 | 26312 | 4,84 | 12 |
|  | HEL | 20q13.33 | 58845445 | 60466282 | 1620837 | 3,89 | 365 |
|  | HL-60 | 20q13.33 | 61458210 | 61551456 | 93246 | 4,36 | 10 |
|  | TF1 | 20q13.33 | 61634576 | 62376959 | 742383 | 3,18 | 69 |

|  | **Cell line** | **Cytoband** | Start | End | Length (bps) | Copy number value | Markers |
| --- | --- | --- | --- | --- | --- | --- | --- |
| D | F-36P | 5q11.1 - 5q11.2 | 48007854 | 53267246 | 5259392 | 1,14 | 558 |
|  | MEG-01 | 5q11.1 - 5q12.3 | 48007854 | 64751326 | 16743472 | 1,27 | 2772 |
|  | MOLM13 | 5q11.2 | 54114560 | 54140718 | 26158 | 1,04 | 10 |
|  | HL-60 | 5q11.2 - 5q15 | 55915416 | 94330201 | 38414785 | 1,26 | 6076 |
| D | F-36P | 9p21.3 | 21437624 | 25424830 | 3987206 | 0,37 | 762 |
|  | EOL-1 | 9p21.3 | 21253887 | 22151353 | 897466 | 0,30 | 214 |
|  | KU-812 | 9p21.3 | 20928278 | 25162803 | 4234525 | 0,69 | 787 |
|  | HEL | 9p21.3 | 20969884 | 24744643 | 3774759 | 0,36 | 704 |
|  | K562 | 9p21.3 | 20735106 | 24705687 | 3970581 | 0,37 | 741 |
|  | NOMO-1 | 9p21.3 | 20461117 | 25147125 | 4686008 | 0,37 | 884 |
|  | MOLM13 | 9p21.3 | 20438426 | 22411876 | 1973450 | 0,75 | 422 |

A: amplification; D: deletion.
