# Supplementary material for: Integration of SNP and mRNA Arrays with MicroRNA Profiling Reveals That MiR-370 Is Upregulated and Targets NF1 in Acute Myeloid Leukemia
Source: PLoS One. 2012 Oct 15;7(10):e47717. doi: 10.1371/journal.pone.0047717 (PMC3471844; doi:10.1371/journal.pone.0047717)
Supplement: Table S2 — List of the high amplifications (CN>5) and homozygous deletions (CN<0.5) found in the copy number analysis of 16 myeloid cell lines. (DOC) [file pone.0047717.s005.doc]

**Supplementary Table 2.** List of the high amplifications (CN>5) and homozygous deletions (CN<0.5) found in the copy number analysis of 16 myeloid cell lines.

| **HIGH AMPLIFICATIONS** | **Cell line** | **start** | **end** | **Cytoband** | **Length (bps)** | **Copy Number  value** | **Markers** |
| --- | --- | --- | --- | --- | --- | --- | --- |
|  |  |  |  |  |  |  |  |
| Chromosome 2 | MEG-01 | 182331491 | 182626451 | 2q31.3 | 294960 | 8,37 | 38 |
|  | MEG-01 | 182736516 | 183111418 | 2q32.1 | 374902 | 8,48 | 81 |
| Chromosome 8 | HL-60 | 130064526 | 130273204 | 8q24.21 | 208678 | 12,81 | 29 |
|  | MEG-01 | 132931702 | 132995383 | 8q24.22 | 63681 | 12,09 | 11 |
| Chromosome 9 | F-36P | 9174669 | 9452892 | 9p23 | 278223 | 8,95 | 89 |
|  | F-36P | 2446040 | 2970007 | 9p24.2 | 523967 | 8,97 | 164 |
|  | HEL | 11306176 | 11390166 | 9p23 | 83990 | 10,32 | 17 |
| Chromosome 13 | F-36P | 70434212 | 71570951 | 13q21.33 | 1136739 | 5,04 | 202 |
|  | F-36P | 81888523 | 83664992 | 13q31.1 | 1776469 | 5,61 | 299 |
|  | F-36P | 86842695 | 87159117 | 13q31.2 | 316422 | 5,20 | 33 |
|  | F-36P | 92221989 | 92381645 | 13q31.3 | 159656 | 5,66 | 31 |
|  | F-36P | 94778104 | 94828355 | 13q32.1 | 50251 | 6,30 | 11 |
|  | F-36P | 98321861 | 98669537 | 13q32.3 | 347676 | 5,25 | 70 |
|  | F-36P | 102720811 | 103149660 | 13q33.1 | 428849 | 5,02 | 144 |
| Chomosome 15 | TF1 | 77804928 | 77828350 | 15q25.1 | 23422 | 17,69 | 10 |
| Chromosome 21 | F-36P | 16507771 | 16586485 | 21q21.1 | 78714 | 27,37 | 25 |
|  | F-36P | 21341239 | 21432066 | 21q21.1 | 90827 | 15,72 | 24 |

| **HOMOZYGOUS DELETIONS** | **Cell line** | **start** | **end** | **Cytoband** | **Length (bps)** | **Copy Number  value** | **Markers** |
| --- | --- | --- | --- | --- | --- | --- | --- |
|  |  |  |  |  |  |  |  |
| Chromosome 2 | EOL-1 | 50629096 | 50933139 | 2p16.3 | 304043 | 0,32 | 59 |
| Chromosome 3 | K562 | 60583442 | 60639341 | 3p14.2 | 55899 | 0,42 | 10 |
| Chromosome 7 | OCI-AML2 | 69369397 | 69498154 | 7q11.22 | 128757 | 0,29 | 14 |
| Chromosome 9 | F-36P | 21437624 | 25773958 | 9p21.3 - 9p21.2 | 4336334 | 0,29 | 686 |
|  | EOL-1 | 21253887 | 22151353 | 9p21.3 | 897466 | 0,30 | 273 |
|  | KU-812 | 21737737 | 28084024 | 9p21.3 - 9p21.2 | 6346287 | 0,30 | 139 |
|  | MOLM13 | 21935211 | 22009401 | 9p21.3 | 74190 | 0,35 | 15 |
|  | NOMO-1 | 20461117 | 31453529 | 9p21.3 - 9p21.1 | 10992412 | 0,28 | 1118 |
|  | HEL | 20969884 | 24744643 | 9p21.3 | 3774759 | 0,27 | 613 |
|  | HEL | 240000 | 1240626 | 9p24.3 | 1000626 | 0,24 | 161 |
| Chromosome 10 | HL-60 | 9066570 | 9105880 | 10p14 | 39310 | 0,3189 | 10 |
|  | HL-60 | 21849531 | 22187383 | 10p12.31 | 337852 | 0,3964 | 10 |
|  | K562 | 103607635 | 103739905 | 10q24.32 | 132270 | 0,3932 | 14 |
