# Supplementary material for: Integration of SNP and mRNA Arrays with MicroRNA Profiling Reveals That MiR-370 Is Upregulated and Targets NF1 in Acute Myeloid Leukemia
Source: PLoS One. 2012 Oct 15;7(10):e47717. doi: 10.1371/journal.pone.0047717 (PMC3471844; doi:10.1371/journal.pone.0047717)
Supplement: Table S3 — MicroRNAs located in amplified regions in myeloid cell lines, which were highly expressed, and had NF1 as a potential target gene. (DOC) [file pone.0047717.s006.doc]

**Supplementary Table 3.** MicroRNAs located in amplified regions in myeloid cell lines, which were highly expressed, and had *NF1* as a potential target gene.

***MicroRNA* p value***

*hsa-miR-370* 0.031

*hsa-mir-379* 0.0001

*hsa-miR-432* 0.050

*hsa-miR-494* 0.0001

**p-value from association analysis between miRNA expression and region with CNV*
