# Supplementary material for: Integration of SNP and mRNA Arrays with MicroRNA Profiling Reveals That MiR-370 Is Upregulated and Targets NF1 in Acute Myeloid Leukemia
Source: PLoS One. 2012 Oct 15;7(10):e47717. doi: 10.1371/journal.pone.0047717 (PMC3471844; doi:10.1371/journal.pone.0047717)
Supplement: Table S4 — NF1 status and miR-370 expression in the 68 samples of patients with AML at diagnosis included in the study. (DOCX) [file pone.0047717.s007.docx]

**Supplementary Table 4.** NF1 status and miR-370 expression in the 68 samples of patients with AML at diagnosis included in the study.

| **Case number** | **NF1**  **(cut-off: ±0.8)** | **NF1 copy number (―ΔCt)** | **NF1 downregulation**  **(cut-off: 2.39)** | **NF1 (―ΔΔCt)** | **miR-370 overexpression**  **(cut-off: 2.62)** | **miR370 (―ΔΔCt)** |
| --- | --- | --- | --- | --- | --- | --- |
| 13676 | N | 0.09 | yes | -4.20 | no | -0.81 |
| 13979 | N | -0.31 | no | -1.44 | no | -4.19 |
| 15109 | N | -0.61 | no | 0.07 | n.d. | n.d. |
| 15619 | n.d. | n.d. | no | -1.26 | n.d. | n.d. |
| 16014 | N | 0.26 | yes | -9.21 | no | -5.18 |
| 17393 | D | -1.00 | no | -0.11 | n.d. | n.d. |
| 17695 | N | -0.26 | no | -1.26 | n.d. | n.d. |
| 18301 | n.d. | n.d. | no | -0.71 | no | 0.36 |
| 18321 | D | -0.93 | yes | -2.74 | no | -3.91 |
| 18425 | N | 0.24 | no | 2.15 | no | -0.43 |
| 18674 | n.d. | n.d. | no | 1.44 | no | 2.07 |
| 18861 | n.d. | n.d. | no | 0.89 | no | 0.47 |
| 18866 | N | 1.18 | no | -1.12 | n.d. | n.d. |
| 19377 | N | 0.83 | yes | -4.19 | yes | 7.22 |
| 19590 | N | 0.48 | no | -2.03 | n.d. | n.d. |
| 19703 | N | 0.12 | yes | -2.29 | yes | 2.80 |
| 19882 | n.d. | n.d. | no | -1.28 | n.d. | n.d. |
| 20492 | n.d. | n.d. | no | -1.72 | no | -1.14 |
| 20531 | n.d. | n.d. | no | 1.52 | no | -3.66 |
| 20762 | D | -1.55 | no | -0.22 | n.d. | n.d. |
| 21065 | N | -0.07 | no | -4.70 | no | -4.78 |
| 21507 | N | 0.05 | yes | -2.54 | yes | 2.79 |
| 21720 | N | 0.94 | no | 1.73 | n.d. | n.d. |
| 23816 | N | 0.20 | yes | -2.49 | n.d. | n.d. |
| 24043 | N | 0.17 | yes | -2.51 | yes | 5.32 |
| 24648 | N | -0.08 | yes | -5.98 | n.d. | n.d. |
| 24731 | D | -1.58 | yes | -2.59 | yes | 2.96 |
| 25222 | N | -0.47 | no | -0.58 | n.d. | n.d. |
| 25352 | N | 0.12 | no | -2.27 | n.d. | n.d. |
| 25413 | n.d. | n.d. | no | -0.62 | no | -7.31 |
| 25604 | D | -1.08 | no | -0.24 | no | -2.70 |
| 25636 | N | 0.40 | yes | -7.11 | yes | 2.70 |
| 25845 | N | -0.03 | yes | -4.02 | n.d. | n.d. |
| 25971 | N | -0.55 | no | 0.34 | no | -1.45 |
| 26059 | n.d. | n.d. | yes | -4.17 | no | 0.47 |
| 26293 | N | 0.37 | no | 0.01 | no | 1.48 |
| 26409 | N | 0.06 | yes | -6.26 | n.d. | n.d. |
| **Case number** | **NF1**  **(cut-off: ±0.8)** | **NF1 copy number (―ΔCt)** | **NF1 downregulation**  **(cut-off:2.39)** | **NF1 (―ΔΔCt)** | **miR-370 overexpression**  **(cut-off: 2.62)** | **miR370 (―ΔΔCt)** |
| 26499 | N | -0.00 | no | 0.53 | no | -4.18 |
| 26806 | n.d. | n.d. | no | -0.73 | n.d. | n.d. |
| 26916 | N | -0.73 | no | -1.14 | no | -1.78 |
| 27101 | N | -0.56 | no | -1.24 | no | -3.13 |
| 27244 | N | -0.46 | no | -2.04 | no | -0.79 |
| 27298 | N | 0.21 | yes | -2.75 | no | -0.35 |
| 27431 | n.d. | n.d. | no | 0.02 | n.d. | n.d. |
| 27988 | N | 0.74 | no | 0.63 | no | -1.90 |
| 27990 | N | 0.02 | yes | -4.89 | no | -1.48 |
| 28383 | N | 0.43 | no | -0.62 | n.d. | n.d. |
| 28399 | n.d. | n.d. | no | -1.23 | no | -3.86 |
| 28591 | n.d. | n.d. | yes | -5.25 | no | -3.12 |
| 29087 | D | -1.32 | no | -0.34 | no | -1.91 |
| 29178 | D | -1.07 | yes | -6.12 | no | -1.77 |
| 29239 | N | 0.21 | no | 0.09 | no | -1.38 |
| 29291 | N | 0.40 | no | 1.84 | no | -1.31 |
| 32014 | N | 0.47 | no | -0.30 | no | -1.41 |
| 38361 | N | -0.25 | no | -0.34 | no | 0.33 |
| 38960 | N | -0.14 | no | 0.41 | no | -0.30 |
| 39742 | D | -1.29 | no | 3.16 | no | -0.89 |
| 40310 | D | -0.98 | no | -0.10 | no | -2.53 |
| 42637 | N | 0.05 | no | 1.88 | no | -1.88 |
| 42386 | N | -0.00 | no | 0.49 | no | -1.05 |
| 42542 | N | -0.38 | no | 1.09 | no | 0.30 |
| 43756 | D | -1.15 | yes | -2.42 | no | -0.55 |
| 44214 | D | -1.93 | yes | -3.07 | no | -1.46 |
| 44325 | N | -0.16 | yes | -2.78 | no | -2.46 |
| 44372 | N | 0.66 | no | 1.50 | no | -3.46 |
| 44459 | D | -1.22 | no | 1.10 | no | -1.09 |
| 44581 | N | -0.40 | no | 1.00 | no | 0.36 |
| 44724 | D | -1.74 | no | 1.36 | no | -1.81 |

**N, normal; D, deletion; n.d., no data.**
