# Supplementary material for: Integration of SNP and mRNA Arrays with MicroRNA Profiling Reveals That MiR-370 Is Upregulated and Targets NF1 in Acute Myeloid Leukemia
Source: PLoS One. 2012 Oct 15;7(10):e47717. doi: 10.1371/journal.pone.0047717 (PMC3471844; doi:10.1371/journal.pone.0047717)
Supplement: Table S5 — Clinical and molecular characteristics of 14 patients with AML at diagnosis included in the study of NF1 at protein level. (DOC) [file pone.0047717.s008.doc]

**Supplementary Table 5.** Clinical and molecular characteristics of 14 patients with AML at diagnosis included in the study of NF1 at protein level.

| **Case** | **Sex** | **Age** | **FAB classification** | **AML type** | **Karyotype** | **NF1 downregulation** | **miR-370 overexpression** |
| --- | --- | --- | --- | --- | --- | --- | --- |
| 1 | Male | 76 | AML-M5 | sAML | 46,XY,t(12;18)(p13;q12)[77%]/ 47,idem,+19[23%] | no | no |
| 2 | Male | 81 | AML-M4 | de novo | 47,XY,+21[93%]/ 46,XY[7%] | no | no |
| 3 | Male | 41 | AML-M4 | de novo | no data | yes | no |
| 4 | Male | 55 | AML-M1 | de novo | 46,XY | yes | yes |
| 5 | Female | 67 | AML-M1 | de novo | 46,XX | yes | no |
| 6 | Female | 64 | AML-M5 | sAML | 46,XX | yes | n.d. |
| 7 | Female | 53 | AML-M4 | de novo | 46,XX | yes | yes |
| 8 | Male | 74 | AML-M5 | de novo | 46,XY | no | no |
| 9 | Male | 78 | AML-M2 | de novo | 46,XY | no | no |
| 10 | Male | 80 | AML-M5 | de novo | 45,X,-Y,dup(7)(q21q32)[83%]/ 46,XY[17%] | no | n.d. |
| 11 | Male | 78 | AML-M5 | de novo | 48,XY,+8,+mar[40%]/ 46,XY[60%] | yes | no |
| 12 | Female | 77 | AML-M1 | de novo | 46,XX | yes | no |
| 13 | Male | 64 | AML-M5 | de novo | 46,XY,t(10;11)(q22;p15) | yes | no |
| 14 | Male | 71 | AML-M1 | sAML | 47,XY,+8 | yes | yes |

sAML: secondary AML; n.d.: no data.
