# Supplementary material for: Integration of SNP and mRNA Arrays with MicroRNA Profiling Reveals That MiR-370 Is Upregulated and Targets NF1 in Acute Myeloid Leukemia
Source: PLoS One. 2012 Oct 15;7(10):e47717. doi: 10.1371/journal.pone.0047717 (PMC3471844; doi:10.1371/journal.pone.0047717)
Supplement: Table S6 — Clinical and molecular characteristics of the 16 human myeloid cell lines. (DOCX) [file pone.0047717.s009.docx]

| **Cell line** | **FAB** | **WHO classification** | **Karyotype** | **N-, K-Ras status** |
| --- | --- | --- | --- | --- |
| HL60 | AML-M2 | AML with maturation | human flat-moded hypotetraploid karyotype with hypodiploid sideline and 1.5% polyploidy - 82-88<4n>XX, -X, -X, -8, -8, -16, -17, -17, +18, +22,+2mar,ins(1;8)(p?31;q24hsr)x2,der(5)t(5;17)(q11;q11)x2,add(6)(q27)x2,der(9)del(9)(p13)t(9;14)(q?22;q?22)x2,der(14)t(9;14)(q?22;q?22)x2, der(16)t(16;17)(q22;q22)x1-2, add(18)(q21) - sideline with: -2, -5, -15, del(11)(q23.1q23.2) - c-myc amplicons present in der(1) and in both markers | Mutant* |
| Kasumi1 | AML-M2 | AML with maturation | human hypodiploid karyotype 45<2n>X, -Y, -9, -13, -16, +3mar, t(8;21)(q22;q22), der(9)t(9;?)(p22;?), der(15)t(?9;15)((?q11;?p11) - carries both partners of 8;21 translocation associated with AML | wt |
| MUTZ3 | AML-M4 | Acute myelomonocytic leukemia | human near-diploid karyotype with 6% tetraploidy; 46(44-48)<2n>XY,t(1;3)(q43;q13)inv(3)(q21q26),t(2;7)(q36;q36)inv(7)(p15q36),t(12;22)(p13;q12); carries t(12;22) recurrent in AML M4; also carries masked inv(3) associated with abnormal megakaryocytopoiesis | wt |
| OCIAML2 | AML-M4 | Acute myelomonocytic leukemia | human hyperdiploid karyotype with 3.8% polyploidy; 48(43-49)<2n>XY,+6,+8,der(1)inv(1)(p36q31)t(1;6)(q13;p12),der(2)t(2;17)(p23;q24.1)del(2)(q14.2q36),der(3)t(1;3)(p36;p25),ins(3;2)(q21;q14.2q36),t(5;8)(q11.2;q24),der(6)t(1;6)(q31;p12)t(3;6)(q26;q24),inv(12)(p13.3q13.2),t(13;14)(q32/33;q24.2),der(17)t(2;17)(p23;q24.1); sideline with +der(5); carries apparent variant translocations involving several ANLL breakpoints: 1p36,3q21,3q26 (megakaryocytic abnormalities),12p13,17q24 | wt |
| MOLM 13 | AML-M5 | Acute monoblastic/acute monocytic leukemia | human hyperdiploid karyotype with 4% polyploidy 51(48-52)<2n>XY, +8, +8, +8, +13, del(8)(p1?p2?), ins(11;9)(q23;p22p23) resembles published karyotype-sideline with idem, +19 -carries occult insertion affecting MLL-AF9 fusion | wt |
| MV411 | AML-M5 | Acute monoblastic/acute monocytic leukemia | human hyperdiploid karyotype 48(46-48)<2n>XY, +8, +18, +19, -21, t(4;11)(q21;q23) | wt |
| NOMO1 | AML-M5 | Acute monoblastic/acute monocytic leukemia | human hyperdiploid karyotype with 8% polyploidy; 46-47<2n>XX,+8,-13,+mar,add(7)(q32),der(9)del(9)(p11p13-21)t(9;11)(p22;q23),t(9;13)(q13;q11),der(11)t(9;11)(p22;q23); carries reciprocal t(9;11) with rearrangement of MLL | Mutant** |
| F36P | AML-M6 | Acute erythroid leukemia | human flat-moded hypodiploid karyotype with 6% polyploidy; 41(40-43)<2n>XY,-3,-13,-16,-19,-21,-22,+2mar, ins(X;13)(p11;q?q?),del(3)(p13p24),add(5)(q11),dup(5)(q11q35),der(6)add(6)(p23)dup(6)(q25q27),der(7)add(7)(p13)add(7)(q12),add(9)(q35),der(9)del(9)(q11q34)inv(9)(p2?4;q34),der(10)t(8;10)(q24;p14)t(10;7)(q25;q12),del(11)(q14q21),der(21)t(10;21)(p14;p12); sideline with dup(1)(p35p36),dup(2)(q1?q2?),del(3)(p13p24) | wt |
| HEL | AML-M6 | Acute erythroid leukemia | human hypotriploid karyotype with 2.3% polyploidy; 63(60-64)<3n>XYY,-2, -9,-10,-10,-11,-14,-16,-16,-17,-19,+20,+21,+2mar,del(2)(q32),t(3;6)(p13;q16),der(5)t(5;17)(q10;q10),der(6)t(1;6)(p13;p21),der(7)add(7)(p14;q32),add(8)(p21), der(9)t(9;?)(?;11)(p24;?)(?;q13),del(11)(q13),add(15)(p11),del(20)(q13),r(20)(p11q11),dup(21)(q11q22.3-qter),psu dic(22;9)t(9;?)(?;22)(p24;?)(?;p11-13); resembles published karyotype; carries also masked 5q- and 20q-consistent with AML M6/MDS | wt |
| KG-1 | AML-M6 | Acute erythroid leukemia | human hypodiploid karyotype with 4.5% polyploidy; 45(42-47)<2n>X/XY,-4,+8,+8,-12,-17,-20,+2mar, der(5;17)(q10;q10)del(5)(q?11q?13),dup(7)(q12q33),del(7)(q22q35),i(8q)x2,der(8)t(6;8)(p11;q22),der(8)t(8;12)(p11;q13),der(11)t(1;11)(q13-21;p11-p13),der(16)t(?12;16)(?p13;q13/21) | wt |
| TF1 | AML-M6 | Acute erythroid leukemia | human highly rearranged hyperdiploid karyotype with 12% polyploidy52-57<2n>XY/XXY,+3,+5,+6,-8,+12,+15,+19,+19,+20,+20,+3mar,der(1)?dup(1)(p21p31)t(1;8)(p36;q11),t(2;12)(q32;q14),t(3;12)(p13-14;p12-13),add(3)(q21),add(5)(q11-13),der(8)t(1;8)(p36;q11),der(12)t(3;12)(p13-14;p12-13)t(1;12)q31-32;q24),add(14)(p12),iso(17)(q10)add(17)(q21),add(19)(q13),trp(19)(q12;q13.3), der(21)t(19;21)(q13.1;q22)dup(19)(q13.1q13.3)t(11;19)(q13;q13.3),der(22)t(19;22)(q11;p11) | wt |
| EOL1 | AML-NOS | acute myeloid (eosinophilic) leukemia | human hyperdiploid karyotype with 7.5% polyploidy; 50(48-51)<2n>XY,+4,+6,+8,+19,del(9)(q22) del(4)(q12) | wt |
| K562 | CML-BP | AML with multilineage dysplasia following MPD (CML BP) | human hypotriploid karyotype without sharp mode; 61-68<3n>XX,-X,-3,+7,-13,-18,+3mar,del(9)(p11/13),der(14)t(14;?)(p11;?),der(17)t(17;?)(p11/13;?),der(?18)t(15;?18)(q21;?q12),del(X)(p22); two markers appear from FISH to have arisen from Ph | wt |
| KU 812 | CML-BP | AML with multilineage dysplasia following MPD | human hypotriploid karyotype with 8% polyploidy; 61(58-62)<3n>XYY,-2,-3,-5,+6,-7,+8,-10,-12,-16,-17,-18,+19,-20,t(9;22)(q34;q11)x2,i(11q),i(17q); Ph (two copies) together with i(17q) and additional chs 8 and 19 | wt |
| KYO-1 | CML-BP | AML with multilineage dysplasia following MPD (CML BP) | 47,XY,6q-,8q+,12p+,+15,15p+,-16,-18,17p+,22p+,22q-(Ph),+22q-(Ph))+15,-16,-18,[C] +15[R] der(?)t(6;12)der(5)t(5;6;12)(?q21;?;?) [R] der(8)T(8;9;17)(q24;?;?)x2 [R] t(3;12)rxp [R] der(11)t(11;17;9)(?q14;?;?)x2 [R] der(9)t(10;9;22)(?;?p11-c-q34;q11)x2 [R] | wt |
| MEG01 | CML-BP | AML with multilineage dysplasia following MPD (CML BP) | human hyperdiploid karyotype with 12% polyploidy; 54(53-56)<2n>XY,+6,+19,+19,+21,+3-4mar,t(1;15)(p13;p13),?inv(3)(p25q26),i(4q),add(5)(p15),der(9)t(9;22)(q34;q11)x2,add(10)(p14),dup(13)(q13q33- 34),add(14)(p11),der(22)t(9;22)(q34;q11); r/dmin x1 present at 57%; sideline with i(5)(q10)del(5)(q11q13) instead of add(5)  Supplementary Table 6. Clinical and molecular characteristics of the 16 human myeloid cell lines. | wt |

*NRAs- exon 3 (c.182A>T), **KRAS exon 2 (c.38G>A)
