# Supplementary material for: Integration of SNP and mRNA Arrays with MicroRNA Profiling Reveals That MiR-370 Is Upregulated and Targets NF1 in Acute Myeloid Leukemia
Source: PLoS One. 2012 Oct 15;7(10):e47717. doi: 10.1371/journal.pone.0047717 (PMC3471844; doi:10.1371/journal.pone.0047717)
Supplement: Table S7 — Oligonucleotide primers used for real-time Q-PCR. (DOC) [file pone.0047717.s010.doc]

**Supplementary Table 7.** Oligonucleotide primers used for real-time Q-PCR.

| **Primer Name** | **Primer sequence** | **Chromosome** | **Amplicon size (bp)** |
| --- | --- | --- | --- |
| G6PDH_Fwd | TCTTCATCACCACAGAGAACT | 1 | 221 |
| G6PDH_Rev | GACCTGGAAGTCACTGGGCA |  |  |
| HEM3_Fwd | TGCACGGCAGCTTAACGAT | 11 | 202 |
| HEM3_Rev | AGGCAAGGCAGTCATCAAG |  |  |
| CLCN7_Fwd | CTCTTAGGCCAGGCGTTTGTG | 16 | 128 |
| CLCN7_Rev | ACCGTGCTCAGCGCTATGC |  |  |
| miR-15a/16_Fwd | TTTATATTCTTTAGGCG | 13 | 291 |
| miR-15/16_Rev | AACACAACTGTAGAGTAT |  |  |
| miR-125a_Fwd | TGCTTTGTCTCAAGAA | 11 | 198 |
| miR-125a_Rev | TACTCAATCACCTCAG |  |  |
| miR-100_Fwd | ATGTCACAGCCCCAAAA | 11 | 131 |
| miR-100_Rev | CCAGGTCCGTGAGATTG |  |  |
| miR-379_Fwd | GTCAGCACCATTCCGTG | 14 | 115 |
| miR-379_Rev | CCAGGTCCGTGAGATTG |  |  |
| miR-494_Fwd | GAATCTTCCTGGAGGT | 14 | 276 |
| miR-494_Rev | GCATGGCACGCTGTCA |  |  |
| miR-370_Fwd | TACAAGTCGGGGCACA | 14 | 107 |
| miR-370_Rev | CTGGTGTTAGACAGAC |  |  |
| miR-127_Fwd | GATGGGTTGACTGATGC | 14 | 164 |
| miR-127_Rev | CGTCGGGCAGTGGAGTG |  |  |
| miR-432_Fwd | GATGGGTTGACTGATGC | 14 | 280 |
| miR-432_Rev | CGTCGGGCAGTGGAGTG |  |  |
